# “Everyone is fighting their own battles”: A qualitative study to explore the context of suicidal ideation among people with HIV (PWH) in Kilimanjaro, Tanzania

**DOI:** 10.1371/journal.pmen.0000318

**Published:** 2025-05-20

**Authors:** Ismail Amiri, Brandon A. Knettel, Clotilda S. Tarimo, Kearsley A. Stewart, Aunchalee E. L. Palmquist, Judith M. Mwobobia, Victor Katiti, Elizabeth Knippler, Linda Minja, Kim Madundo, Elizabeth F. Msoka, Alyssa Martinez, Judith Boshe, Michael V. Relf, Blandina T. Mmbaga, David B. Goldston

**Affiliations:** 1 Duke Global Health Institute, Duke University, Durham, North Carolina, United States of America; 2 Kilimanjaro Christian Medical Centre, Moshi, Tanzania; 3 Duke University School of Nursing, Durham, North Carolina, United States of America; 4 Duke Center for Global Mental Health, Durham, North Carolina, United States of America; 5 Brown School, Washington University in St. Louis, St. Louis, Missouri, United States of America; 6 Duke Center for AIDS Research, Duke University, Durham, North Carolina, United States of America; 7 Kilimanjaro Clinical Research Institute, Moshi, Tanzania; 8 Kilimanjaro Christian Medical University College, Moshi, Tanzania; 9 Department of Psychiatry and Behavioral Sciences, Duke University, Durham, North Carolina, United States of America; Ss Cyril and Methodius University in Skopje: Saints Cyril and Methodius University in Skopje, NORTH MACEDONIA

## Abstract

Tanzania faces significant HIV-related challenges with 1.4 million people currently living with HIV, 33,000 new infections, and 22,000 AIDS-related deaths annually. Suicide is a leading cause of death among People with HIV (PWH), with one-quarter of all deaths by suicide in Tanzania occurring among PWH. Despite this challenge, mental health resources are scarce, with only 55 psychologists and psychiatrists in the country, and clinic staff in HIV care lack adequate mental health training. This qualitative study explores the experiences of PWH who have recently had suicidal thoughts. The aim is to create targeted mental health interventions in Kilimanjaro. Participants were screened for suicidal ideation during routine care at two HIV clinics, with semi-structured qualitative interviews conducted thereafter. Data were analyzed using thematic analysis aided by NVivo 12 software. PWH experiencing suicidal ideation encounter multiple stressors related to their HIV diagnosis, societal stigma, financial stress, and broader social challenges. Suicide is sometimes viewed as an escape from these difficulties. Coping mechanisms include seeking assistance from family and religious leaders, but social support is hindered by fear of stigma. While participants expressed openness to counseling, treatment options were extremely limited. Suicide risk among PWH is influenced by stressors related to HIV, such as socioeconomic challenges, HIV stigma, low social support, and accompanying psychological distress. There is a clear need for improved mental health care options customized to the needs of PWH in Tanzania and other low-resource settings.

## Introduction

Annually, over 700,000 individuals die by suicide, with the majority coming from low- and middle-income countries [1]. Globally, suicide is a leading cause of death among people with HIV (PWH) [2–4], and in Tanzania, more than one-quarter of all deaths by suicide are among PWH [5,6]. Tanzania also has only 55 psychiatrists and psychologists in a nation of more than 63 million people, with a clear gap between the demand for mental health services and the available care [7,8].

Suicide prevention efforts in Tanzania remain limited [9]. There are no national suicide prevention programs [9], and existing mental health services are often restricted to major urban centers [10]. General healthcare providers, who frequently lack specialized mental health training, are the main source of psycho-social support [11]. While some non-government organizations (NGOs) offer mental health services, these are not widely accessible. The Tanzanian government has taken some steps toward improving mental health care, such as integrating mental health in to primary care settings and training community health workers in psychological first aid but these initiatives remain underdeveloped and underfunded. The absence of a structured suicide prevention framework leaves a critical gap in care for vulnerable population, particularly PWH. The availability of mental health medications is limited, and medications are not affordable for most people [12].

Further, existing services may be ineffective in addressing suicidal ideation because of a lack of understanding of its complexity, including the role of impulsivity versus planning, decision- making, and comorbid mental disorders (CMDs) [13]. Suicidal ideation is not a singular phenomenon but rather a complex experience shaped by various psychological and social determinants [14]. Decision making in suicidal behavior often reflects a complex interplay between impulsivity and planning [15]. Some individuals experience acute emotional distress. Leading to impulsive acts, while others engage in prolonged planning, weighing their options over time [16]. These tendencies can be exacerbated by CMDs such as depression, anxiety and substance use disorders, which impair cognitive flexibility and increase feelings of hopelessness [17]. Additionally, loneliness and social isolation are both highly prevalent among PWH and can reinforce negative thought patterns, reducing perceived social support and increasing vulnerability to suicidal ideation and behavior [18].

Suicidal behavior is both illegal and also deeply stigmatized in Tanzania; therefore, many incidents go unreported [19]. The social, cultural, and legal environment of silence around suicide creates barriers for individuals and families who might hesitate to disclose or discuss suicide-related incidents [20]. Tanzanians with HIV face multiple challenges, including physical challenges related to HIV stigma [5]. and various symptoms of depression and anxiety, these symptoms can originate from a range of sources and contribute to emotional distress and broader social challenges [21–23]. In Tanzania, the COVID-19 pandemic further complicated the landscape, with early evidence suggesting a rise in suicide rates during this period [24].

Socioeconomic factors such as poverty and unemployment further intensify the mental health crisis among PWH in Tanzania. An estimated 26.4% of Tanzanians live below the world poverty threshold, defined as living on less than $1.90 a day [25], and as of 2023, the nation grapples with unemployment rates of 2.6% [24]. pushing many into informal sectors without job security. HIV stigma and other challenges unique to PWH may further contribute to socioeconomic difficulties in this population [26], as people with HIV may experience work-related discrimination or unemployment [27]. These economic struggles, combined with challenges like limited healthcare access, contribute to the elevated stress and risk of suicidal ideation among PWH [27]. Gender differences also play role, with more men likely to die by suicide while women tend to report more attempt [28,29],reflecting different patterns of suicidal behavior that warrant tailored strategies.

Misconceptions around HIV, such as the belief that HIV is a death sentence or that transmission to others is likely, can also fuel discrimination, isolation, loss of support, and heightened feelings of shame and hopelessness, reinforcing a cycle of mental health risk [19,30]. Together, shared risk factors and social determinants of poor health combine to create a complex pattern of closely interrelated psychological, social and economic challenges that drive suicidal ideation and behavior, making intervention challenging.

We are seeking to develop a sustainable, culturally sensitive, and effective counseling intervention to reduce suicidal ideation among people with HIV (PWH) in Kilimanjaro, Tanzania. The *IDEAS* for Hope intervention, which we are preparing to implement, is rooted in principles of motivational interviewing-enhanced safety planning (MI-Safe Cope) and problem-solving therapy to address social determinants of health and reduce stigma [31–33]. The *IDEAS* for hope intervention was developed through a multi-step process incorporating both global evidence-based strategies and local contextual adaptation [34]. Initially we conducted a literature review of effective suicide prevention intervention among PWH in Africa [35].We then held consultations with Tanzanian mental health providers, community leaders, and individual with lived experiences of HIV and suicidality to ensure cultural and contextual relevance. Based on these insights, we adapted a Motivational Interviewing-enhancing safety Planning (MI-Safe Cope) [36] and problem solving Therapy [37], to address the unique challenges faced by PWH in Kilimanjaro. The intervention was Piloted in a small feasibility study, which informed further refinements before broader implementation [33]. The intervention empowers participants to identify their strengths, coping mechanisms, and support networks while collaboratively developing strategies to navigate challenges and enhance their sense of hope and resilience [38]. By integrating evidence-based approaches into the intervention framework, we aim to address the complex interaction of emotional distress and HIV-related challenges comprehensively.

The objectives of this study were to conduct qualitative interviews to explore the personal experiences of PWH who have reported recent suicidal ideation, shedding light on the intricate aspects of their mental health challenges, and (B) to gain insights that can inform future interventions and treatment strategies tailored for this critically underserved and highly stigmatized population.

## Materials and methods

### Ethics statement

All study procedures received approval from the Tanzanian National Institute of Medical Research (Approval: NIMR/HQ/R.8a/VOL.IX/3782) and the ethical review boards of the Duke University Health System (Protocol ID: Pro00107424) and Kilimanjaro Christian Medical Center (KCMC), (Proposal number 1307). Additionally, we obtained formal approval from the regional and district health authorities, who serves as representative of the local community and provided permission of the study to be conducted in their region. These approvals ensured alignment with local policies and community interests. All participants provided written informed consent before they agreed to participate in this study, including explicit consent to have the interviews recorded.

From 27^th^ September, 2021–26^th^ September 2022, we conducted semi-structured in-depth qualitative interviews with 15 PWH in Kilimanjaro, Tanzania who were experiencing suicidal ideation. This research took place at two adult HIV Care and Treatment Centers (CTCs): Kilimanjaro Christian Medical Center (KCMC) and Pasua Health Center. KCMC, which is a larger facility, caters to a significant patient load of 900–1200 individuals monthly for regular HIV care appointments. On the other hand, Pasua Health Center, which serves approximately 200 patients monthly, is a smaller-scale facility. In Tanzania, HIV care is provided for free to all individuals attending government hospitals [39].

Fifteen participants were randomly selected to complete in-depth qualitative interviews, nine from KCMC and six from Pasua, out of a sample of 80 participants enrolled in a larger study focused on the validation of measures of suicide risk among adults living with HIV in Tanzania [33,40]. All participants from the larger validation study [40], were adults attending routine HIV care appointments who reported experiencing suicidal ideation in the past 30 days. Additionally, they needed to be physically and cognitively capable of providing informed consent and completing the study procedures.

HIV clinic nurses were trained specifically to screen all of the adult patients for suicidal ideation during routine HIV care appointments using a single yes/no question derived from the Columbia Suicide Severity Rating Scale [39], translated into Kiswahili: “In the last month, have you had actual thoughts of killing yourself?”(Kiswahili: *Kwa kipindi cha mwezi mmoja uliopita umewahi kupata mawazo ya kujiua?).*The training focused on how to ask a brief, three-question screening tool, which included two depression-related questions and one suicide related question. The nurses were trained on how to compassionately and effectively ask these questions to ensure accurate responses. A total of 3885 participants were screened; 80 (2.1%) of them screened yes for suicidal ideation and enrolled in the larger study [40].

After the screening, participants who endorsed suicidal ideation underwent a more in-depth risk assessment. This assessment was conducted by trained research staff, including individuals with experience in mental health counseling. The assessment went beyond the initial screening to evaluate the present of active suicidal intent, providing a comprehensive understanding of an individual’s risk. Individuals who exhibited active suicidal intent or high-risk behavior were promptly referred to a mental health specialist within the healthcare system for immediate support. This system aimed to ensure safety while also identifying factors contributing to suicidal ideation.

The research assistant identified 15 participants for this qualitative sub-study by selecting every 4th participant. Interested individuals were informed of the sub-study and accompanied to a private room to meet with I.A., a trained research assistant with a Bachelor’s degree and extensive experience in qualitative research. The research assistant read aloud the informed consent, allowing participants to ask questions before obtaining their written consent, and conducted semi-structured qualitative interviews in the local language (Kiswahili). We conducted preliminary analyses during data collection and established that data saturation occurred after 15 interviews as no new themes emerged [41].

Our interview guide was carefully created to explore the experiences and challenges faced by PWH, including emotional well-being, medication adherence, stigma, and suicidal ideation, ensuring a comprehensive understanding of their situation. By including probing questions, we aimed to capture the complexity of their experiences and emerging themes that arose during the interviews. For additional details, see S1 Table: Summary of Interview Guide. All interviews were audio-recorded and audio files were transferred to a secure electronic database. Audio recordings of in-depth interviews were transcribed verbatim in Swahili, translated to English, and de-identified by a bilingual team member (I.A & C.T). Thematic analysis of the transcribed data followed six steps [42]: (1) Familiarization of data in initial readthrough of the transcripts, (2) Generation of initial inductive codes, with two team members (B.K and I.A), coding two transcripts each, (3) Combining codes into themes in a team-based approach until consensus was reached on a code book structure, (4) Reviewing themes as additional transcripts were coded to revise and finalize the code book, (5) Determining the significance of themes based on the final coding, and (6) Reporting of findings. All data underwent coding utilizing NVivo 12 software [43]. For quality assurance, four interview transcripts (27%) were randomly double-coded by a second evaluator (B.K) and assessed for inter-coder agreement with a predetermined benchmark of 80% agreement. This process ensured satisfactory coding quality and reproducibility [44]. Interrater reliability for the selected interviews was calculated above the acceptable benchmark, with a range of 82.4-86.3% agreement.

After the interview was completed, all participants received compensation of 10,000 Tanzanian shillings (approximately USD 4.50) for their time. Further, all participants received a brief safety planning intervention using the procedures outlined by Stanley and Brown [45], as well as referral information for standard-of-care counseling services [5]. Participants with an active plan or intent to attempt suicide were linked directly to a mental health worker at the hospital by the research assistant.

## Results

The participants in the study had a median age of 42 years and the majority (n = 13, 87%) were female. Most (n = 12, 80%) had attended secondary school, and relationship status varied widely (see Table 1).

**Table 1:** Demographic information of study participants.

Age	N (%)
•18-28	3 (20%)
•29-39	3 (20%)
•40-50	8 (53%)
•51 and over	1 (7%)
Gender	
•Male	2 (13%)
•Female	13 (87%)
Education	
•Grade 7 or above	12 (80%)
•Less than Grade 7	3 (20%)
Relationship Status	
•Married	5 (33%)
•In a relationship	1 (7%)
•Single	2 (13%)
•Divorced	4 (27%)
•Widowed	3 (20%)

Prevailing themes from the interviews included descriptions of the participant’s journey of living with HIV, experiences of suicidal ideation and drivers of mental health challenges, social determinants of suicidal ideation, experiences of mental health treatment and coping, and feedback on a potential intervention model to address suicidal ideation in HIV care. See Table 2 for sub-themes that emerged under each theme and representative quotations for these themes.

**Table 2:** Themes related to suicidal ideation among people with HIV in Kilimanjaro, Tanzania.

Main Themes	Sub-Themes	Representative Quotes
HIV journey	*- HIV testing and diagnosis* - HIV stigma - Medication adherence	*“Hmm, I felt incredibly distressed because I wasn’t anticipating receiving such news, especially considering where I was in life at that moment. It hit me hard.”*
Suicidal ideation and drivers of mental health challenges	*- Suicide attempt* - HIV Infection and associated health challenges - Family conflicts, isolation, and stigma	*“I swallowed all the pills with a glass of water, but I didn’t feel any different until my neighbor returned and asked what I was doing. I confessed how angry my husband had made me. All she found were the empty blister packs, so she hurried to the shop and brought back some milk.”*
Social determinants of suicidal ideation	*- Financial stress* - Challenges with housing - Food insecurity	*“Without health insurance, I am left untreated and financially burdened when illness strikes, making me feel hopeless about my future.”*
Mental health treatment and coping skills.	*- Religious coping* - Family &friends - HIV clinic staff - Financial support &work opportunity	*“I don’t know, I just heard a voice in my soul telling me not to kill myself, to stay there, and to pray to God to help me.”*
Feedback on IDEAS for Hope Counseling Intervention	*- Suggestions for intervention content* *- Addressing HIV stigma* *- Building hope and reasons for living*	*“I think it will be ok if they will be able to get counseling like what you gave me today.”* *“You can also tell them not to worry about anyone who may stigmatize them because life is more important than what anyone else is thinking of them.”*

### HIV Journey

In discussing their experiences with HIV, participants shared details about the events leading up to their decision to get tested, the day they were tested and diagnosed with HIV, and their experiences after the diagnosis. They talked about their deliberations on whether to disclose their status to family, their feelings about the stigma associated with HIV, and their adherence to HIV medication. Participants shared poignant stories about the testing and diagnosis process, highlighting the difficulty in deciding to get tested and the challenges they faced upon receiving a positive diagnosis, often unexpectedly. For example, a 42-year-old woman recounted the emotional turmoil of being diagnosed with HIV during her pregnancy, describing the distress she initially felt, her concerns about her own and her unborn child’s health, and the dilemma of disclosing her status to her husband and others.

*“It was discovered* [that I was living with HIV] *when I was pregnant with my first child. To tell the truth, I felt very bad and I cried a lot. Even though I was quick to say that I wanted to know about my health condition, frankly, that was one of my worst days. Where was I going to start? What was I going to do? When I got home, how could I tell my husband? I felt like I should die.”* (Female, 42 years).

When asked about HIV stigma or mistreatment, participants shared experiences of enacted stigma (i.e., stigmatizing actions received from others), feelings of self-blame and shame directed toward themselves (internalized stigma), and fears of stigma (anticipated stigma) that might occur if their HIV status were to become known by more people. Out of the 15 participants, 12 expressed how difficult it was when people treated them badly because they lived with HIV, which in some cases directly contributed to thoughts about suicide.

One woman shared, *“It is very painful* [to live with HIV] *and it makes me feel very weak. I feel as though I am completely valueless. All the time, I think ‘It would be better for me to die. There is no point in continuing to live like this.’ It is as if I mean nothing.”* (Female,28 years).

In addition to the feeling of self-blame and shame shared by participants, it is also important to understand how they personally attributed their HIV diagnosis. Majority of participants openly described the internalized stigma they experienced, where feeling of worthlessness was a recurring them. While some participants expressed self-blame for contacting the virus, others pointed to external factors such as lack of awareness or blaming their partners behavior as a contributing factor, as one woman said *“I blame myself for not being so careful with him, as we had intimate while he was drunk and carelessly”* (Female 28 Years).

Although not directly addressed by all participants, the relationship between HIV disclosure and violence raised as an important area of concern. Several participants experienced violence from their partners as a response to sharing their HIV status, which made it even more difficult for them to disclose their status to other family members. One woman shared *“I was not feeling free to share with my family because when I shared with my husband, he blamed me and bitten me, saying that I planned to kill him”* (Female 43 Years)

All but one of the participants (93%) reported consistently adhering to their HIV medication at the time of the interview; however, one person reported adherence challenges. *“Yes, I completely refused to use the medications for five years because my CD4 was high. A time came when they told me that I needed to start the antiretrovirals since my CD4 count was decreasing. I became very sick and I was admitted here.”* (Female,49 years).

### Suicidal Ideation and Drivers of Mental Health Challenges

We also inquired about the personal experiences of individuals living with HIV in Kilimanjaro, Tanzania. A common experience shared by all participants was openly discussing feelings of hopelessness and considering suicide. For instance, one woman revealed that she had purchased rat poison with the intention of taking her own life, but ultimately decided against it after praying*. “I have considered suicide many times. I said, ‘Today I must finish myself off. I *don’t *want to continue like this.’ There was a day when I went to buy rat poison. I put it in a cup and went outside while the children were sleeping. I cried a lot and prayed to God to forgive me. I told Him that I wasn’t doing this because I wanted to, but because the things in my life were difficult. Whatever I was doing failed and my life was moving backward.”* (Female, 42years).

Suicide attempts were common among the participants, with 4 out of 15 having made such attempts. As an example, a 25-year-old woman, who was diagnosed with HIV when she was a young child, decided to end her life by taking pills during a moment of intense sadness. “*I just decided to take a bunch of pills and finish my life off, and as I took the medications, I just managed to swallow two pills. When I was onto the third one, my sister found me and noticed that I was behaving strangely.”* (Female,25years).

Common factors associated with mental health challenges and suicidal thinking included the stress of living with HIV, financial challenges, family conflict, and stigma**.** The emotional challenges caused by HIV infection continually emerged as a key burden. A significant number of participants described experiencing symptoms of depression and anxiety linked to their HIV status, as well as serious social challenges, that combined to contribute to feelings of hopelessness and suicidal ideation. One woman shared that she was depressed because she did not know where she contracted the HIV infection and was experiencing stigma from her family members.

*“What depressed me is that no one could tell me how I got HIV. It causes me so much pain because my relatives regard me as an outcast.”* (Female, 50 years).

### Social determinants of health and suicidal ideation

The social determinants of health have emerged as significant issues linked to suicidal thoughts. These include financial stress, family conflict, housing insecurity, and food insecurity. People suffering from depression and suicidal thoughts often face practical challenges such as a lack of money. For example, one person shared how a health crisis, worsened by limited financial resources, led to emotional distress. This highlights the difficulties individuals with HIV may encounter within the healthcare system, particularly in areas with limited resources where financial challenges are common, even without accompanying health issues.

*“I was once sick and I *didn’t *have any money. I came here and I was supposed to pay for treatment. I was coughing a lot, I had chest tightness, and I *wasn’t *feeling well. I was supposed to pay for lab tests but I didn’t have any money.”* (Female, 39 years).

Family conflict emerged as a significant factor associated with suicidal ideation, with a majority of respondents expressing those conflicts within their families added to their distress. One instance involves a 42-year-old woman who, upon sharing her suicidal thoughts with a family member, received an un-supportive and negative response.

*“Someone said, ‘If you want to die, just kill yourself. No one has any need for you and it is only the children who will suffer. So, if you want to kill yourself, go ahead.’ I just left and went home.”* (Female, 42 years).

Challenges associated with living arrangements posed a significant source of mental stress for most participants. More than half participants either lacked proper housing or had to reside with relatives, sharing rooms with others, including children. This situation made it challenging for them to use their HIV medication due to a lack of privacy.

*“Life is a very long journey. Living with family members is a challenge because I live with their children in their rented house where I share a room with the children. Therefore, these caused me stress and I said to myself, ‘Oh my God, help me to get my room so that I can live on my own and be free.”* (Female,28years).

Many People with HIV face challenges accessing enough healthy food, and some struggle with thoughts of suicide. It’s important for people with HIV to take their medication with food to reduce nausea and other side effects. However, many participants had trouble finding food to take with their medication. One person even mentioned being unable to provide for their child, which took a toll on their mental and emotional well-being. *“My child was hungry and I *didn’t *know what to do. So, I thought it would be better to kill myself. So that was the reason.”* (Female,39 years).

### Mental health treatment and coping strategies

When asked about their mental health coping strategies, few participants had sought formal mental health treatment such as counseling or psychiatric services. Instead, they reported seeking support from alternative sources, including seeking guidance and reassurance from religious leaders and confiding in HIV clinic staff. For those who did not have anyone to talk to, some turned to coping through substance abuse. More than half of the participants had spoken to a religious leader, asking for prayers and advice as a primary means of addressing their mental health-related challenges, including thoughts of suicide. *“I recently approached the pastor seeking assistance regarding suicidal thoughts. Initially, the pastor shared passages from the Bible with me, emphasizing the gravity of suicide as a significant sin under any circumstances.”* (Female, 42 years).

With few options for formal mental health treatment available, several participants reported confiding in HIV clinic nurses when they experienced mental health challenges. Counseling provided by HIV nurses was generally restricted to education about HIV and treatment but sometimes would extend to conversations about emotional health and social stressors that were driving suicidal thinking. *“When I have deep thoughts and feel unable to solve them myself, I always seek help from nurses. The service providers at the CTC counseled me and introduced me to individuals who had children and families, showing me how they lived their lives* (Male,47 years).

Upon encountering mental health challenges, a few participants turned to using substances such as alcohol as a coping mechanism. *“That’s my only way of reducing stress when I get stressed out by him running around with different women. So, I just go and drink. I feel like I do not have another option*. (Female, 45 years).

### Feedback on the ideas for hope counseling intervention

To develop tailored mental health interventions in Kilimanjaro, participants were asked to provide input on the development of the Ideas for Hope counseling intervention. This intervention will involve screening all HIV care patients for suicidal thoughts and providing telehealth-delivered counseling sessions. Participants gave feedback on the proposed intervention content, the acceptability of the model, and potential challenges that could arise. For instance, participants stressed the importance of HIV education, including informing patients that HIV is not immediately life-threatening when adhering to antiretroviral medication. They also emphasized the critical role of medication adherence in maintaining long-term physical and emotional health, as well as daily functioning*. “You should tell* [patients] *that HIV is not a disease that can kill unless you neglect the medicine. If you take your medicine, you will be well, your work will go on, and your family will grow”.* (Female, 52 years).

Concerning strategies to reduce the burden of HIV stigma, participants emphasized the importance of maintaining a positive mindset, prioritizing a healthy lifestyle, and adhering to medication, which can empower individuals to lead long and fulfilling lives. *“Therefore, individuals should be encouraged to stop focusing on the disease as a limitation and instead pursue their dreams and objectives with vigor. When society focuses on the disease, it becomes a burden to those affected. It is very important to address and eliminate the stigma surrounding HIV. Allowing an individual to set their objectives and focus on these without societal judgment. By doing this we empower those living with HIV to lead full, meaningful lives, free from the weight of stigma”.* (Female,28 years).

Participants highlighted the importance of reassuring individuals who are newly diagnosed with HIV and fostering a sense of hope for the future. *“When someone is newly diagnosed with HIV, they need to be reminded that it is not the end of the world and that there is still hope for tomorrow. Medications can help them to live a normal life”.* (Male,28 years).

## Discussion

This study investigated the impact of various psychosocial factors on suicidal thoughts among People with HIV in Kilimanjaro, Tanzania. Our findings demonstrated that stigma, financial stress, and family conflicts have a significant effect on the mental well-being of participants. The emotional strain of receiving an HIV diagnosis, compounded by societal stigma and financial hardships, notably contributed to suicidal thoughts among our participants. These findings are consistent with existing research that delves into the intricate relationship between an HIV diagnosis, mental health issues [46], and the societal factors influencing individuals’ coping mechanisms [47,48]. Our study emphasizes the pressing need for integrated mental health care within HIV treatment strategies to reduce the risk of suicide and support the overall well-being of those affected.

Our study findings support the importance of adopting a holistic approach to HIV care that extends beyond the traditional biomedical model by attending to patients’ emotional health [49]. Integrating mental health screening and treatment into routine HIV care can serve as a crucial tool in identifying individuals at risk of suicidal ideation and providing targeted support [33,40]. Moreover, interventions should prioritize addressing the emotional impact of an HIV diagnosis, combating stigma, and equipping individuals with adaptive coping strategies to navigate the multifaceted challenges they face [22]. By acknowledging and addressing the complex psychosocial factors influencing mental well-being among individuals living with HIV, we can work towards more comprehensive and effective interventions that support their overall health and quality of life [49].

Social determinants, particularly poverty and resulting struggles with housing and food insecurity, play a crucial role in shaping mental health outcomes. For example, poverty has been linked to higher risk and poorer outcomes for both HIV and mental health challenges, including increased risk for suicide attempts and death by suicide [50]. Similarly, our study highlights that housing challenges, food insecurity, and family conflicts are significantly associated with suicidal ideation among individuals with HIV in Kilimanjaro. When children are involved, this can serve as a protective factor for suicide due to concerns about leaving the children behind, but can also serve as an additional stressor due to worry about not being able to provide for children’s basic needs. Thus, there is a clear need for comprehensive interventions that address not only the physical and mental health of the individual but also underlying socioeconomic challenges that affect the entire family [51]. We recommend efforts to reduce suicidal ideation extend beyond clinics, involving collaborative initiatives with healthcare, social services, and community organizations. Housing assistance, financial support, and community-based interventions are vital in creating a supportive environment that tackles broader structural issues affecting mental health and HIV outcomes [52].

Our findings shed light on the utilization of alternative support systems, particularly religious leaders, for mental health treatment and coping skills among individuals living with HIV in Kilimanjaro. Participants often sought comfort and support through religious guidance and prayers to manage their mental health struggles, including suicidal thoughts. This underscores the cultural and spiritual dimensions influencing coping within this population. Notably, a study conducted in the United States revealed a significant negative association between religiosity and suicidal thoughts, suggesting there is potential for religious leaders to support interventions for suicidal thoughts [53]. Collaborative models that integrate both formal mental health services and community-based support, such as religious leaders, can enhance the accessibility and acceptability of mental health care for individuals living with HIV.

We actively involved religious leaders in our proposed intervention and during the implementation phase. They were included in our implementors meetings, where they provided very useful feedback on how they can support the intervention. These leaders expressed willingness to be part of the process, offering their role in educating their congregation and providing emotional and spiritual support. However, it is important to acknowledge that data on religion and coping may be mixed, sometimes resulting in increased stigma depending on the views of the church, because people with suicidal thoughts may be considered sinners and religion may be a potential source of stigma [54,55]. Thus, it is important to ensure that religious leaders are well informed about mental health to avoid perpetuating harmful stigma and instead foster an environment of support.

This study did not measure or focus on the religious background of individuals, However the population in Kilimanjaro is predominantly Christian, with a significant Muslim minority and participants in this study followed this religious affiliation. As we plan to expand our intervention, we will ensure that we engage religious leaders from both Christian and Muslim communities to ensure inclusivity and cultural relevance.

Our sample included older adult, which aligns with demographic trends of individuals seeking HIV care in the Kilimanjaro region. While our intervention was not specifically focused on the youth population, we acknowledge the concerning trend of higher suicide rates among younger individuals. As we look ahead to future studies, we plan to explore how our intervention, especially the telehealth component, can be adapted for younger populations. Given the growing prominence of telehealth among youth, this platform holds potential for addressing youth suicide risks and providing accessible mental health support tailored to their unique needs.

Hope emerged as a crucial theme in our findings, with many participants identified hope as a key factor in their ability to cope with the challenges associated with Living with HIV. While our current study did not quantitatively measure hope, we recognize its importance and plan to include it as an outcome variable in future phases of our intervention. Assessing levels of hope before and after the intervention will provide valuable insight in to the effectiveness of our approach and its potential to influence participants’ mental health and overall well-being.

The strengths of this study lie in its qualitative approach, allowing for in-depth exploration of the personal experiences and perspectives of individuals living with HIV and experiencing suicidal ideation in the Kilimanjaro region of Tanzania. We elicited diverse narratives and used direct quotes to enhance the authenticity and richness of the findings. Moreover, the feedback on the IDEAS for Hope counseling intervention provides practical insights for developing a mental health intervention, directly informed by people with personal experience. Limitations of the study include its cross-sectional design, which hinders the ability to establish causation or observe changes over time. We obtained valuable qualitative data; however, we acknowledge that the perspectives of these 15 individuals in a single region of the country may not be representative of broader populations. Further, the reliance on self-reported experiences introduces potential recall bias. Additionally, there is a noted gender imbalance in the sample, as our study included only two men out of fifteen systematically selected participants. However, this gender breakdown is representative of the individuals seeking HIV care who endorsed suicidal ideation and were eligible for the study.

This study incorporated qualitative findings into the IDEAS for Hope counseling intervention, which include universal screening for suicidal ideation in routine HIV, followed by telehealth-delivered counseling sessions [33]. Participants highlighted the importance of HIV education in fostering hope, promoting medication adherence, and reducing HIV-related stigma [56].While a U.S study found strong support for telehealth interventions [56], these models remain untested in African settings [35,57]. This underscores the significant, yet untapped, potential of telehealth in expanding mental health care access for PWH in Tanzania.

## Conclusions

This study highlights the urgent mental health issues faced by People with HIV (PWH) in Kilimanjaro, Tanzania. It emphasizes the need for comprehensive and culturally sensitive interventions. It is important to integrate mental health screening into routine HIV care, address social factors contributing to suicidal thoughts, and use culturally relevant coping strategies to improve mental health outcomes. The positive feedback on the proposed Ideas for Hope counseling intervention indicates the potential of universal screening and telehealth-delivered counseling. The next steps involve implementing these findings into testable interventions and actionable policies, working with stakeholders to integrate evidence-based mental health interventions into HIV care, and continuously assessing and refining interventions through ongoing research. The comprehensive approach outlined in this research lays the groundwork for creating a more supportive and resilient environment for People with HIV in Kilimanjaro and beyond.

## Supporting information

S1 Table Summary of the semi-structured interview guide used during in-depth interviews with PWH in Kilimanjaro Tanzania. The guide includes initial open-ended questions and follow-up probes exploring participant’s experiences with HIV diagnosis, treatment adherence, emotional wellbeing, suicidal ideation, stigma and perspective on a potential counseling intervention.
(DOCX)
