# Supplementary material for: “Everyone is fighting their own battles”: A qualitative study to explore the context of suicidal ideation among people with HIV (PWH) in Kilimanjaro, Tanzania
Source: PLOS Ment Health. 2025 May 20;2(5):e0000318. doi: 10.1371/journal.pmen.0000318 (PMC12798187; doi:10.1371/journal.pmen.0000318)
Supplement: S1 Table — The guide includes initial open-ended questions and follow-up probes exploring participant’s experiences with HIV diagnosis, treatment adherence, emotional wellbeing, suicidal ideation, stigma and perspective on a potential counseling intervention. (DOCX) [file pmen.0000318.s001.docx]

S1_Table. Summary of Semi-structured Interview Guide

| Summary of Semi-structured Interview Guide | |
| --- | --- |
| **Initial questions** | **Additional probes** |
| 1. Can you share with me what it was like for you when you were told you had HIV? | When were you first diagnosed? |
| 1. Can you tell me about any challenges you've faced in taking ARVs consistently? | What were some reasons for these challenges? |
| 1. How confident do you feel about taking HIV medication moving forward? | Are there reasons you might stop the medication? |
| 1. Have you experienced any feelings of sadness, stress, or worry related to your HIV status? | What caused those feelings? |
| 1. Have you ever thought about harming yourself or ending your life since being diagnosed with HIV? | How often have you felt this way? When was the last time you felt this way? |
| 1. If you had these thoughts, who did you speak to? How did this help (if at all)? | What types of support would help to keep you safe if you ever had these thoughts again? |
| 1. Have you ever experienced stigma or mistreatment from anyone in your life because you are living with HIV? | [If yes] What did this look like? How did you feel? |
| 1. What topics or messages do you think would be important to include in a counseling intervention for people with HIV who are struggling with thoughts of ending their lives? |  |
| 1. Do you think patients would be comfortable with having counseling phone calls in a private office at the HIV clinic? | How about having counseling over WhatsApp? |
| 1. If you were meeting with a counselor via WhatsApp, how many sessions would you like to have? | How long would you like the sessions to be? |
| 1. If you had the opportunity to participate in this type of intervention, would you be interested? | How do you think it would help? |
| 1. What concerns or suggestions do you have about an intervention like this? |  |
